# Mycotic pseudoaneurysms complicating renal transplantation: a case series and review of literature

**DOI:** 10.1186/1752-1947-6-59

**Published:** 2012-02-14

**Authors:** Polytimi Leonardou, Sofia Gioldasi, George Zavos, Paris Pappas

**Affiliations:** 1 Department of Radiology, Laikon General Hospital of Athens, 17 Ag. Thoma str., 115 27Athens, Greece

**Keywords:** renal transplantation, iliac artery pseudoaneurysm, mycotic aneurysm, percutaneous treatment

## Abstract

**Introduction:**

Kidney transplantation can be complicated by infection and subsequent development of mycotic aneurysm, endangering the survival of the graft and the patient. Management of this condition in five cases is discussed, accompanied by a review of the relevant literature.

**Case presentations:**

Five patients, three men 42-, 67- and 57-years-old and two women 55- and 21-years-old (mean age of 48 years), all Caucasians, developed a mycotic aneurysm in the region of the anastomosis between renal graft artery and iliac axes. Four patients presented with systemic fever and iliac fossa pain and one presented with hemorrhagic shock. Morphologic investigation by color doppler ultrasonography revealed a pseudoaneurysm at the anastomotic site. A combination of antibiotic therapy, surgery and interventional procedures was required as all kidney transplants had to be removed. No recurrence was recorded during the follow-up period.

**Conclusions:**

A high index of suspicion is required for the timely diagnosis of a mycotic aneurysm; aggressive treatment with cover stents and/or surgical excision is necessary in order to prevent potentially fatal complications.

## Introduction

The incidence of mycotic aneurysm formation following renal transplantation is < 1%. A non-mycotic etiology for aneurysm formation in the same setting has also been recorded [1-3]. An aneurysm may be asymptomatic, being an incidental finding during imaging evaluation for other reasons, yet it can occasionally cause fever and anemia, iliac fossa discomfort, renal dysfunction and graft loss or even precipitate a lethal hemorrhage due to acute rupture [4]. Early recognition of this entity based on a high index of suspicion and use of early diagnostic procedures is vital for its successful management. Therapeutic options include aneurysmectomy, proximal and distal ligation of the arterial trunk, medical treatment and percutaneous embolization, although the choice of prompt treatment strategy is still a subject of debate, requiring further delineation.

We report the cases of five patients suffering from this life-threatening complication and present the therapeutic procedures used and their long-term outcomes together with a literature review on this topic.

## Case presentations

### Case one

**Figure 1:**
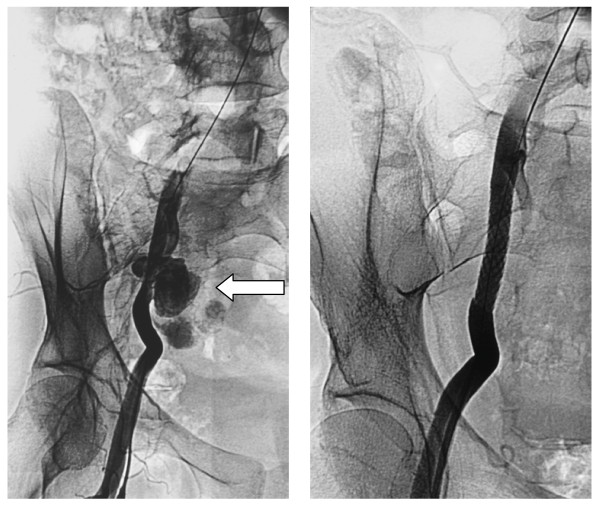
**Mycotic pseudoaneurysm's (arrow left) exclusion with covered stent (right)**.

**Figure 2:**
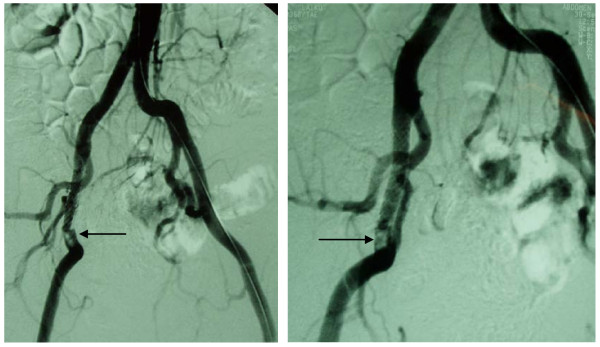
**(Same patient as Figure 1) Fresh thrombus at the distal part of the covered stent (arrows), placed to exclude the pseudoaneurysm at the site of renal transplant arterial ligation**. This thrombus proved to be infected by mucorales hyphae.

The first case is a 42-year-old Caucasian man, who was on hemodialysis after rejection of a kidney allograft of unknown etiology four years ago. Three months after nephrectomy, the patient was admitted to our hospital with diffuse pain in his right lower quadrant, acute ischemia of his right lower limb with the presence of petechiae and a systemic temperature of 38°C. Angiographic investigation revealed a pseudoaneurysm at the site of the previous arterial ligation and three balloon expandable cover stents were deployed during the same procedure to exclude the aneurysm (Figure 1). Following this interventional procedure, the patient was continuously febrile (38.5°C) and his blood examinations revealed leukocytosis. He then developed clinical signs of thromboembolism, which were confirmed by angiography (Figure 2). Subsequent treatment involved embolectomy and the histological examination of the thrombus identified mucorales hyphae. Histological examination also revealed mucorales infection of the stents. The patient underwent surgery for removal of both the mycotic aneurysm and the stents, and for construction of a suprapubic femoral-femoral by-pass. He also received amphotericin B for three months. No recurrence or any other major complication has been recorded during a follow-up period of eight years and he was able later on to undergo a kidney transplantation successfully.

### Cases two and three

One man, 67-years-old, and one woman, 55-years-old, both Caucasian, had previously received a transplant from cadaveric donors, and presented with fever and abdominal pain located at the left iliac fossa. Color doppler ultrasonography (CDU) examination revealed the presence of a hypoechoic mass at the hilus of the transplanted kidney with bidirectional or swirling blood flow within its lumen.

**Figure 3:**
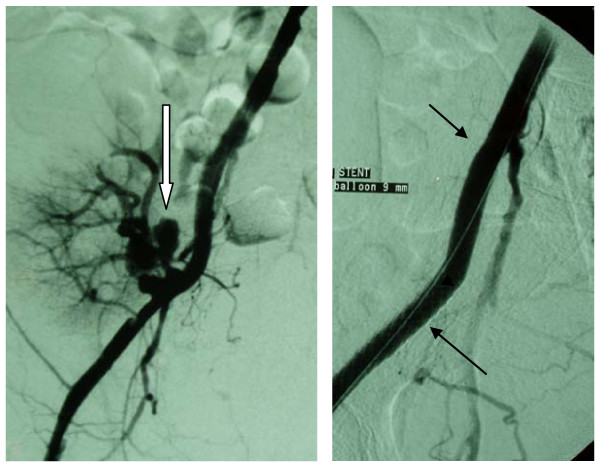
**Angiographic image before (arrow left) and after covered stent placement (arrows right) for exclusion of mycotic pseudoaneurysms arising from the transplant renal arterial bed**.

In both cases the blood cultures grew pseudomonas aeruoginosa. Both patients received ciprofloxacin intravenously for eight days and then orally for another three weeks. The subsequent angiographic investigation revealed a pseudoaneurysm formation (Figure 3). They were both treated with covered stent insertion followed later on by nephrectomy. They remain on hemodialysis without any reoccurrence of vascular infection evident on regular examination. The follow-up period ranges from 18 up to 36 months.

### Case four

**Figure 4:**
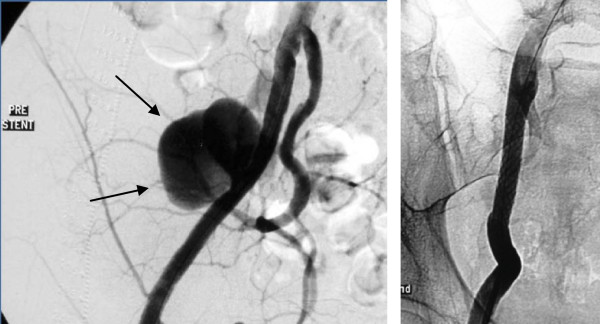
**Post transplantectomy pseudoaneurysm (arrows) of the external iliac artery before (left) and after (right) cover stent placement**.

A 57-year-old Caucasian man developed a pseudoaneurysm three months after transplantectomy, at the site of the former transplant artery ligation. He presented with signs of right lower quadrant pain and tenderness, fever and weakness. CDU examination revealed a pseudoaneurysm. Klebsiella pneumoniae was isolated from blood cultures. He received intravenous antibiotic therapy (colistin) and a week later two balloon expandable covered stents were placed to exclude the aneurysmal sac from the external iliac artery (Figure 4). He was discharged home without complications and was living independently at his 14-month follow-up.

### Case five

**Figure 5:**
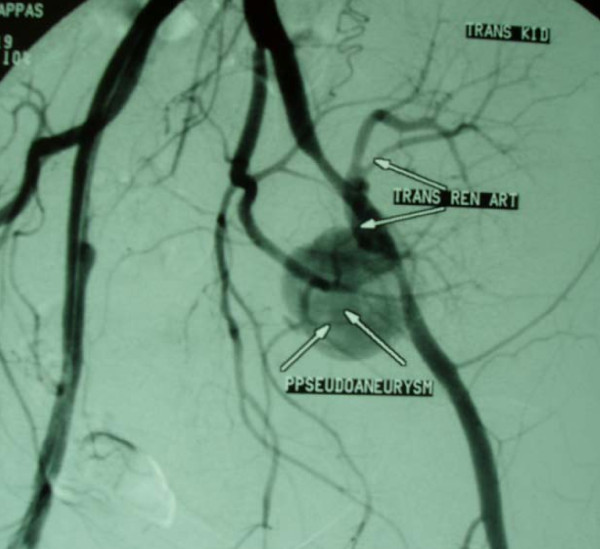
**Ruptured mycotic pseudoaneurysm (arrows pseudoaneurysm) at the site of anastomosis between iliac and transplant renal artery (arrows transplant renal artery)**.

**Figure 6:**
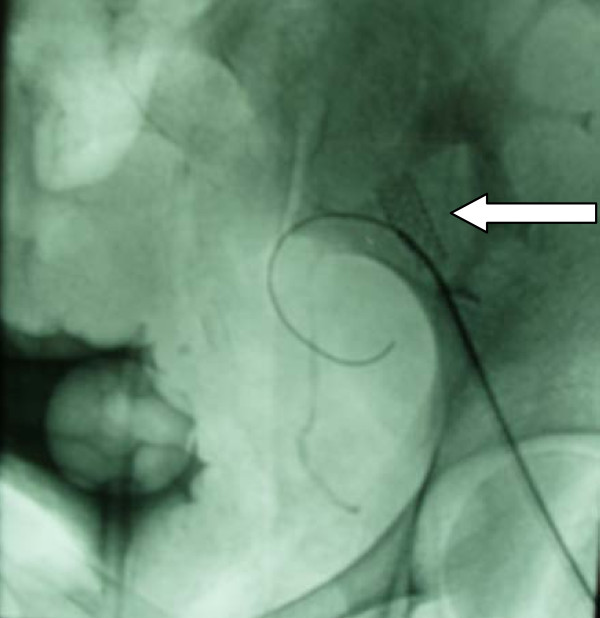
**Neck modification of the aneurysm with a bare stent (arrow) before coil embolization**.

**Figure 7:**
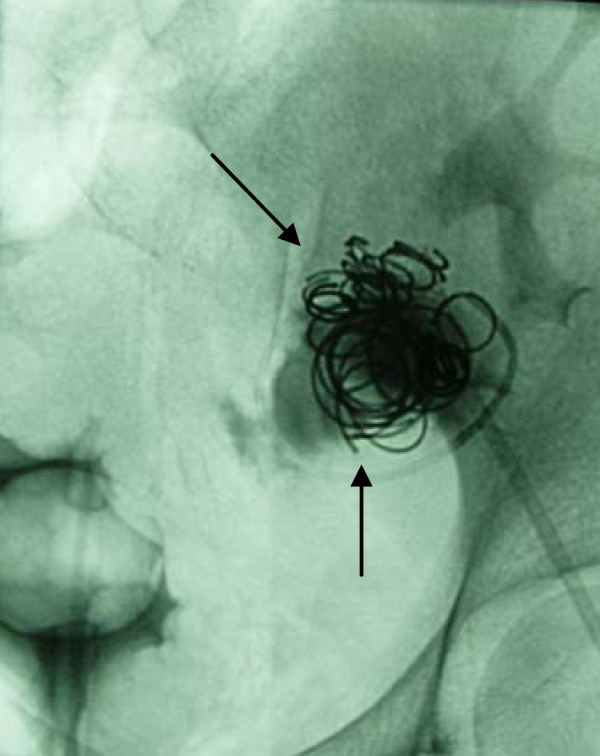
**Aneurysmal coil packing (arrows)**.

**Figure 8:**
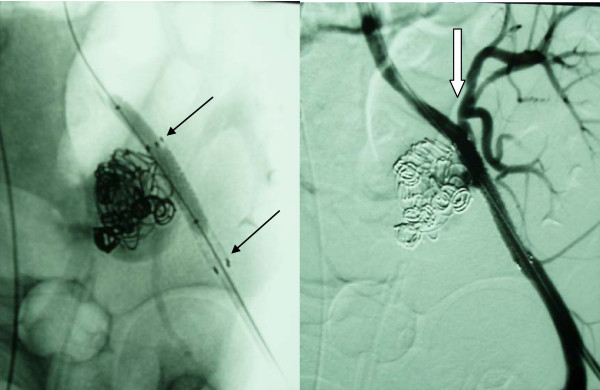
**Deployment of a covered stent close to the aneurysmal neck (arrows left) with preservation of the transplant renal artery (arrow right)**.

**Figure 9:**
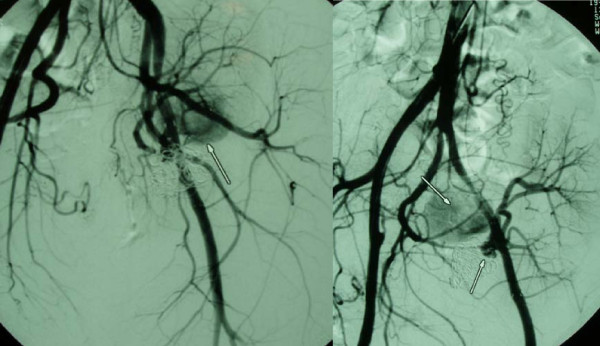
**Clinical symptoms and pseudoaneurysm revealed by Color Doppler Ultrasonography reappeared some days later and was verified by angiography (arrows)**.

**Figure 10:**
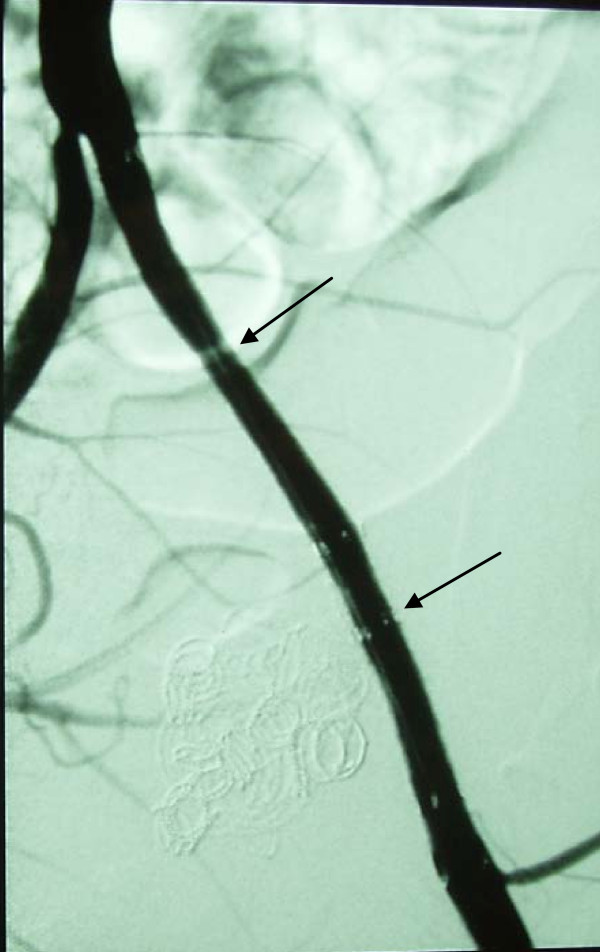
**New covered stent deployment at the orifice of the transplant renal artery (arrows) with sacrifice of the transplanted kidney, which was removed surgically some days later**.

The fifth patient was a 21-year-old Caucasian woman, who had received a renal transplant from a living donor 15 months previously and subsequently developed a mycotic pseudoaneurysm. Apart from iliac fossa pain and fever, there were also signs of abdominal hemorrhage. CDU examination revealed the presence of an aneurysm at the anastomotic region and retroperitoneal blood collection. Blood cultures grew Candida which was treated with intravenous liposomal amphotericin. Percutaneous transluminal treatment was suggested and the pseudoaneurysm was initially packed with coils followed by placement of a covered stent, aiming to keep patent the transplant renal artery (Figures 5, 6, 7, 8). After a short, clinically stable, postoperative period, reappearance of a pseudoaneurysm was recorded (Figure 9); this was treated by insertion of a second covered stent (Figure 10) and renal transplant removal with successful surgical restoration of iliac arteries. Antifungal therapy with oral fluconazole had been continued for two months.

## Discussion

Infection of the artery at or around the site of anastomosis is an ominous complication commonly presenting as an anastomotic leak or local dissolution of the arterial wall [5]. Mycotic aneurysm is a rare infectious complication, associated with high morbidity and mortality rates, while the prompt treatment modality remains a subject of debate. Small, asymptomatic pseudoaneurysms can be managed conservatively by regular monitoring, while early diagnosis and timely operation might be the most important factors in the survival of patients with mycotic pseudoaneurysms [6,7]. However, an aneurysm infected with candida is reported to have been successfully managed by conservative pharmacological means during a period of 38 months follow-up [8].

Another point of interest is the need for nephrectomy in the majority of renal transplant patients presenting with pseudoaneurysm due to resistant rejection, function failure or infection. A review of the literature found reports of successful venous patch angioplasty and surgical reconstruction of a renal artery aneurysm that prevented aneurysm rupture and saved the graft [9,10]. With regard to our fourth patient, although we decided on a two-stage procedure aiming to preserve the transplanted kidney, this was not feasible due to recurrence of the pseudoaneurysm.

Another aspect of significant consideration is the need for concomitant vascular reconstruction after surgical aneurysmectomy. As Bracale *et al*. [11] suggest, after investigation of 11 transplant recipients with mycotic aneurysm formation, vascular reconstruction is advisable since it prevents lower limb ischemia. Moreover, an above-knee amputation was reported in a 25-year-old woman who developed foot ischemia three months after external iliac ligation [2].

With regard to our cases, survival of the first patient was achieved due to a multi-disciplinary therapeutic approach involving medical and surgical interventions. There are published reports that support the efficacy of only one treatment modality (either pharmacological or interventional). On the other hand Liapis *et al*. [12], presented a study in which 11 out of 17 patients with mucorales infection who underwent surgery survived, while the remaining ones who did not have surgery died, thus reinforcing the view that medical therapy alone is not sufficient. We agree with Liapis *et al*. and remain sceptical of the proposed pathway of a single treatment modality [13,14].

## Conclusions

Taking into account the serious complications that can arise from mycotic aneurysm we feel that increased awareness and close monitoring are indispensable. The benefits derived from early diagnosis and treatment are indisputable. However, as a prompt therapeutic strategy has not been delineated yet, prospective studies are essential for thorough evaluation of treatment options.

## Consent

Written informed consent was obtained from the patients for publication of these case reports and any accompanying images. Copies of the written consents are available for review by the Editor-in-Chief of this journal.

## Competing interests

The authors declare that they have no competing interests.

## Authors' contributions

PP and GZ were in charge of patient management for the surgical procedures. PL and SG were major contributors to the writing of the manuscript. All authors read and approved the final manuscript.
